# Removal of Crystal Violet by Using Reduced-Graphene-Oxide-Supported Bimetallic Fe/Ni Nanoparticles (rGO/Fe/Ni): Application of Artificial Intelligence Modeling for the Optimization Process

**DOI:** 10.3390/ma11050865

**Published:** 2018-05-22

**Authors:** Wenqian Ruan, Jiwei Hu, Jimei Qi, Yu Hou, Rensheng Cao, Xionghui Wei

**Affiliations:** 1 Guizhou Provincial Key Laboratory for Information Systems of Mountainous Areas and Protection of Ecological Environment, Guizhou Normal University, Guiyang 550001, China; qianwenruan@163.com (W.R.); qqijimei@163.com (J.Q.); 15887298226@163.com (Y.H.); 18230825324@163.com (R.C.); 2 Cultivation Base of Guizhou National Key Laboratory of Mountainous Karst Eco-environment, Guizhou Normal University, Guiyang 550001, China; 3 Department of Applied Chemistry, College of Chemistry and Molecular Engineering, Peking University, Beijing 100871, China; xhwei@pku.edu.cn

**Keywords:** crystal violet, graphene, bimetallic Fe/Ni nanoparticles, artificial intelligence, zero point of charge

## Abstract

Reduced-graphene-oxide-supported bimetallic Fe/Ni nanoparticles were synthesized in this study for the removal of crystal violet (CV) dye from aqueous solutions. This material was characterized by X-ray diffraction (XRD), scanning electron microscopy (SEM) coupled with energy dispersive spectroscopy (EDS), Raman spectroscopy, N_2_-sorption, and X-ray photoelectron spectroscopy (XPS). The influence of independent parameters (namely, initial dye concentration, initial pH, contact time, and temperature) on the removal efficiency were investigated via Box–Behnken design (BBD). Artificial intelligence (i.e., artificial neural network, genetic algorithm, and particle swarm optimization) was used to optimize and predict the optimum conditions and obtain the maximum removal efficiency. The zero point of charge (pH_ZPC_) of rGO/Fe/Ni composites was determined by using the salt addition method. The experimental equilibrium data were fitted well to the Freundlich model for the evaluation of the actual behavior of CV adsorption, and the maximum adsorption capacity was estimated as 2000.00 mg/g. The kinetic study discloses that the adsorption processes can be satisfactorily described by the pseudo-second-order model. The values of Gibbs free energy change (Δ*G*^0^), entropy change (Δ*S*^0^), and enthalpy change (Δ*H*^0^) demonstrate the spontaneous and endothermic nature of the adsorption of CV onto rGO/Fe/Ni composites.

## 1. Introduction

Crystal violet (CV) as a cationic dye belongs to the class of triphenylmethane dyes, which is used for different purposes, such as dermatological agents, biological staining, textile dying, and paper printing [1,2,3]. Colored dyes create issues in the ecosystem as they are not only nonbiodegradable, toxic, mutagenic, and carcinogenic but also reduce light penetration and affect the photosynthetic activity of aquatic life [4,5,6]. CV is commonly stable when discharged into waste water owing to the complex aromatic molecular structure; therefore, it is imperative to remove this dye from waste water [7,8]. There are many methods for the removal of dyes like biological treatment, oxidation, photochemical degradation, membrane separation, coagulation, and adsorption [9]. However, adsorption has been adopted as a superior method for the removal of dyes because of the advantages such as low cost, ease of operation, and good efficiency.

**Figure 1:**
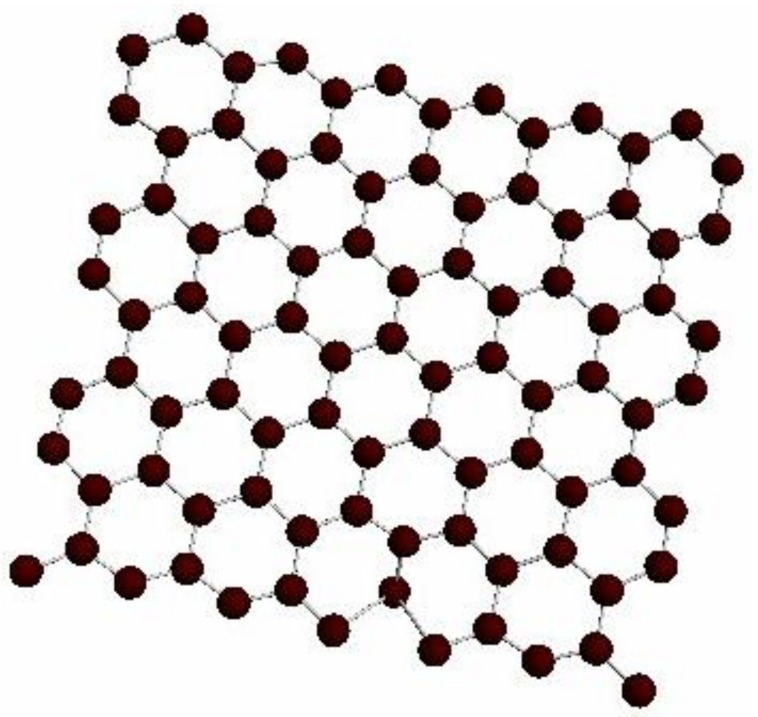
Model structure of graphene oxide (GO).

Nanoscale zero-valent iron (nZVI) with small particle size (1 to 100 nm), can be obtained through the sonochemistry method, electrochemical method, and the liquid-phase or gas-phase reduction methods. Recently, nZVI has gained interest as a more promising material for the long term because of its large specific surface area and high reactivity, and this material has been utilized for the remediation of wastewater contaminated with heavy metals, halogenated organic compounds, dyes, and phenol [10,11,12,13,14]. Nevertheless, nZVI can be oxidized and aggregated in air; as a result, it will render a lower reactivity and removal efficiency [15]. A further effort to improve the performance of nZVI was combination with a second metal (such as Ni, Pd, or Pt) which has been reported to enhance the dechlorination rate of chlorinated hydrocarbons. However, such bimetallic nanoparticles are still susceptible to several drawbacks, e.g., strong tendency to be oxidized, aggregated, and corroded during the dechlorination process. Recently, graphene oxide (GO), containing a range of reactive oxygen functional groups, has attracted multidisciplinary interest due to its excellent electrical, mechanical, and thermal properties (Figure 1) [16,17,18], and it has been applied in the fields of sensors, field-effect transistors, polymer composites, and nanocomposites [17]. Fe adsorption on graphene has been investigated previously by using computational simulation techniques [19,20,21,22,23]. Moreover, reduced graphene oxide (rGO) has high chemical stability, which is a good alternative as the support. rGO has been successfully used to immobilize nZVI for photodegradation of chlorophenols and the removal of heavy metals and dyes. Artificial intelligence (AI) techniques, such as artificial neural networks (ANNs), genetic algorithms (GAs), particle swarm optimization (PSO), adaptive neuro fuzzy inference systems (ANFISs), and support vector machines (SVMs), have been extensively used for modeling of the adsorption processes [24,25,26]. AI techniques have been applied in various fields, e.g., automatic programming, big data, pattern recognition, intelligent internet search, image understanding, autonomous driving, robotics, and human–computer games [27]. An ANN is constructed taking inspiration from the biological neurons in the human brain, which can solve complex and nonlinear problems with suitable amount of data, but its main disadvantage is that the solutions are easily trapped in a local optimum [28,29]. Both PSO and GA are powerful population-based techniques for optimizing problems to avoid a local optimum.

In this work, response surface methodology (RSM), ANN-GA, and ANN-PSO were applied to optimize and predict the process conditions for the maximum removal efficiency of CV removal from aqueous solutions. Parameters investigated for the CV removal include the effect of initial dye concentration, initial pH, contact time, and temperature. The rGO/Fe/Ni composites were synthesized by the co-precipitation method and characterized though X-ray diffraction (XRD), scanning electron microscopy (SEM) in conjunction with energy dispersive spectroscopy (EDS), Raman spectroscopy, N_2_-sorption, and X-ray photoelectron spectroscopy (XPS). The zero point of charge (pH_ZPC_) of rGO/Fe/Ni composites was determined by using the salt addition method. The isotherm models of Freundlich, Langmuir, Temkin, and Dubinin–Radushkevich (D-R) were adopted to analyze the experimental data. Adsorption kinetics were examined by using pseudo-first-order, pseudo-second-order, intraparticle diffusion, and Elovich models. In addition, thermodynamics parameters (Gibbs free energy change, entropy and enthalpy changes) were calculated using the Van’t Hoff equation.

## 2. Experimental Section

### 2.1. Materials

**Figure 2:**
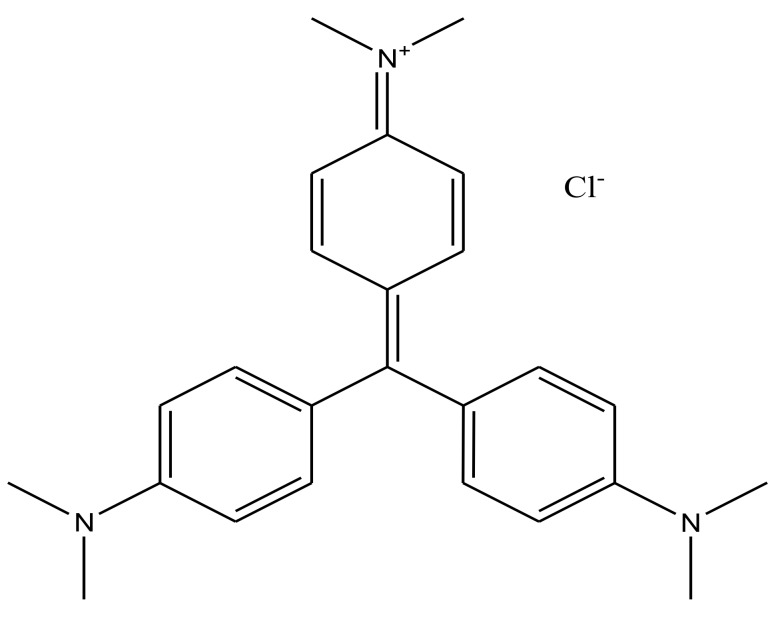
Structural formula of crystal violet (CV).

All reagents and chemicals used in this work were of analytical grade, including H_2_SO_4,_ FeSO_4_·7H_2_O, NiCl·6H_2_O, NaBH_4_, HCl, and NaOH. Crystal violet (molecular formula: C_25_H_30_N_3_Cl, molecular weight = 408 g/mol, λ_max_ = 583 nm) used in this work was supplied by Tianjin Kemio Chemical Co., Tianjin, China (Figure 2, Table 1). The stock solution of this dye (1000 mg/L) was prepared with deionized water. Graphite powder (particle diameters < 30 μm) was purchased from Sinopharm Chemical Reagent (Beijing, China).

### 2.2. Preparation of the Nanomaterials

#### 2.2.1. Synthesis of GO

GO was synthesized by the modified Hummers method [30]. Quantities of 2.0 g graphite powder and 0.5 g NaNO_3_ were placed into 40 mL H_2_SO_4_ in a 500 mL beaker under continuous stirring. Then, 5.0 g KMnO_4_ was slowly added and stirred for 2 h below 20 °C. After this, the temperature of the solution was raised to 35 °C and kept for 30 min during the above-mentioned process. Subsequently, the reaction mixture was heated to 98 °C and allowed to react for 15 min under stirring. Finally, H_2_O_2_ (30 wt %) was added to the reaction mixture, and the yellow-brown graphite oxide solution was washed several times with diluted HCl (5 wt %) and deionized water. The resultant nanoparticles were obtained by centrifugation, then dried at 60 °C for 48 h in vacuum.

#### 2.2.2. Synthesis of Fe/Ni Nanoparticles and rGO/Fe/Ni Composites

The rGO/Fe/Ni composites were synthesized by the co-precipitation method [31]. FeSO_4_·7H_2_O solution and NiCl·6H_2_O solution were added into GO solution using ultrasonication for about 2 h. The mixture was then stirred for 12 h. After this, 5.2 g NaBH_4_ dissolved in 50 mL of deionized water was added to the mixture. The black precipitate was obtained via centrifugation with three deionized water and ethanol washing cycles, and then dried at 50 °C under a vacuum for 24 h before characterization. Furthermore, Fe/Ni nanoparticles were also prepared similarly to rGO/Fe/Ni composites without adding the GO.

### 2.3. Characterization of the Prepared Nanomaterials

X-ray diffraction patterns of Fe/Ni and rGO/Fe/Ni were obtained using a Philips Analytical X-ray (Lelyweg 1 7602, EA, Almelo, The Netherlands) with a Cu Kα X-ray source (generator tension 40 kV, current 40 mA) in the range of 5–90°. The morphology and dimensions of these materials were characterized by scanning electron microscopy (Quanta F250, FEI, Hillsboro, OR, USA) coupled with energy dispersive spectroscopy. Raman measurements were performed by using LabRAM HR800 spectroscopy recorded at a 532 nm laser source (Horiba Jobin Yvon, Paris, France). The specific surface areas of Fe/Ni nanoparticles and rGO/Fe/Ni composites were determined using the N_2_ adsorption/desorption isotherms at 77 K (Brunauer–Emmett–Teller (BET) Quadrasorb SI, Quantachrome Instruments, Boynton Beach, FL, USA). The Fe/Ni and rGO/Fe/Ni composites were characterized by X-ray photoelectron spectroscopy using an ESCALAB 250Xi spectrometer (Thermo Electron Corporation, Waltham, MA, USA).

### 2.4. Determination of the Zero Point of Charge

The pH of an aqueous solution is an important factor that may influence the adsorption process. The zero point of charge (ZPC) is defined as the pH value where a net surface charge equal to zero is indicated [32]. The pH_ZPC_ of rGO/Fe/Ni composites was determined by using the salt addition method [33]. A quantity of 30 mL of NaCl (0.05 mol/L) solution was added to several 100 mL Erlenmeyer flasks. Initial pH (pH_i_) values of NaCl solutions were adjusted over a range from 2 to 10 by adding 0.1 mol/L HCl and NaOH. pH_i_ values of solutions were then accurately recorded, and 50 mg of each adsorbent was added to each flask. Suspensions were shaken at 298 K for 48 h. The suspensions were centrifuged at 4500 rpm for 5 min, and the final pH (pH_f_) values of the suspensions were recorded. The value of pH_ZPC_ is the point where the curve of ΔpH (pH_f_–pH_i_) versus pH_i_ crosses the line equal to zero while pH_ZPC_ was determined by the intersection point of the curve.

### 2.5. Experiments

The removal of CV by rGO/Fe/Ni composites was studied in a batch system. Conical flasks with volume 100 mL were used for mixing 20 mg rGO/Fe/Ni with 50 mL of solution of known CV concentration, initial pH, temperature, and contact time. The solutions were agitated with a thermostatically controlled shaker at 200 rpm. The initial pH values were adjusted by the addition of 0.1 mol/L HCl or 0.1 mol/L NaOH to conduct the batch experiments at the desired pH. The isotherm study was carried out with different initial concentrations of CV from 200 to 1000 mg/L, keeping the other variables constant. The kinetic study was done by varying time from 5 to 24 min. For the thermodynamic study, the temperature was varied from 298 to 318 K. Then, the adsorbents were separated by centrifugation, and the final concentration of CV was analyzed by measuring a UV-visible spectrophotometer at λ_max_ of 583 nm. The percentage removal (*Y*) and the amount of CV removal at equilibrium, *q_e_* (mg/g), were calculated by the following equations:

(1) $$Y=\frac{\left(C_{i}-C_{f}\right)}{C_{i}}\times 100\%$$

(2) $$q_{e}=\frac{(C_{i}-C_{f})\times v}{m}$$

where *C_i_* (mg/L) and *C_f_* (mg/L) are the initial and final CV concentrations in solution, respectively; *v* is the volume of the solution (mL); and *m* is the dosage of the adsorbent (mg).

### 2.6. Optimization of Operating Parameters

#### 2.6.1. Box–Behnken Design (BBD)

The Box–Behnken Design (BBD), as one of the designs for experiments of response surface methodology (RSM), was used to investigate the combined effects of independent variables, namely, initial dye concentration (*X*_1_), initial pH (*X*_2_), contact time (*X*_3_), and temperature (*X*_4_), with the minimum number of combinations for the four factors mentioned above [34]. A total of 29 experiments, involving the four operating parameters at three levels and five replications of the central point, were devised by BBD. This model will also give the maximum removal efficiency of CV under optimum conditions. Each independent variable has 3 levels designated as −1, 0, and +1 for low, middle, and high values, respectively (Table 2). The relationship of the independent variables and the percentage decolorization of CV is described by the second-order polynomial

(3) $$Y=\beta_{0}+\beta_{1}X_{1}+\beta_{2}X_{2}+\beta_{3}X_{3}+\beta_{4}X_{4}+\beta_{5}X_{1}X_{2}+\beta_{6}X_{1}X_{3}+\beta_{7}X_{1}X_{4}+\beta_{8}X_{2}X_{3}+\beta_{9}X_{2}X_{4}+\beta_{10}X_{3}X_{4}+\beta_{11}X_{1}{}^{2}+\beta_{12}X_{2}{}^{2}+\beta_{13}X_{3}{}^{2}+\beta_{14}X_{4}{}^{2}$$

where *Y* is the removal efficiency of CV; *X_i_* (*i* = 1–4) are noncoded variables; and *β_j_* (*j* = 0–14) are the regression coefficients for intercept, linear, quadratic, and interaction effects, respectively (*i* ≠ *j*).

#### 2.6.2. ANN Modeling

**Figure 3:**
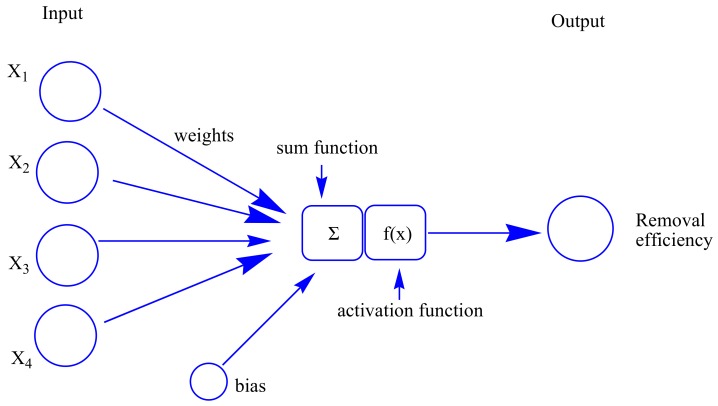
Schematic for the artificial neuron model.

ANNs are capable of machine learning and pattern recognition, which can solve problems like learning, thinking, remembering, and reasoning. The artificial neurons are the basic elements of ANNs, and consist of many simple computational elements that are connected to each other. The “network” is defined as the structure in which the neurons act simultaneously in a group. In the present study, a three-layer feed-forward perceptron ANNs with a back-propagation (BP) algorithm was established for modeling purposes [35]. This network consists of an input layer, hidden layers, and an output layer. All input weights were summed to create the output through the activation function [36] (Figure 3). The tangent sigmoid transfer function (tansig) (Equation (7)) was used in the input–hidden layer, whereas the linear transfer function (purelin) (Equation (8)) was adopted in the output layer.

All the input and output were normalized within uniform range (0.1–0.9) during training of the network. Normalization of the input data was done using the following equation:

(4) $$x_{i}=2\times \left(x-x_{\min}\right)/\left(x_{\max}-x_{\min}\right)-1$$

where *x_i_* is the corresponding scaled variable for an input variable (*x*); likewise, *x*_min_ and *x*_max_ are the minimum and maximum values of the variable, respectively.

(5) $$W_{i}=\sum_{i=1}^{k}w_{ij}x_{i}$$

(6) $$sum=W_{i}+\theta$$

In Equations (5) and (6), *x_i_* is the value of a neuron in the input layer, *w_ij_* is the corresponding connection weight between neuron *i* in the input layer and neuron *j* in the hidden layer, *W_i_* is the connection weight, and *θ* is called the bias. The tangent sigmoid (tansig) function and linear transfer function (purelin) were used between the input and hidden layers and between the hidden and output layers, respectively.

(7) $$f\left(x\right)=2/\left(1+e^{-2x}\right)-1$$

(8) f(x)=x

The output was produced by the weight and bias of neurons through the activation function using the following equation:

(9) Y=f×sum

where *Y*, *f*, and *sum* represent the output, activation function, and all weights and biases in hidden layer or output layer, respectively.

The relative influence of the individual variable was calculated by the following Garson equation [37,38]:

(10) $$I_{ab}=\frac{\sum_{e}^{n}\left(\frac{\left|w_{ae}\right|}{\sum_{g}^{m}\left|w_{ge}\right|}\left|w_{eb}\right|\right)}{\sum_{z}^{n}\left(\sum_{l}^{n}\left(\frac{\left|w_{al}\right|}{\sum_{g}^{m}w_{ge}}\left|w_{eb}\right|\right)\right)}$$

where *I_ab_* is the relative importance of the *j*th input variable to the output variable; *w_x_* is the connection weight; and *a*, *e*, and *b* are the number of neurons in the input layer, hidden layer, and output layer, respectively.

#### 2.6.3. Optimization Using ANN-GA and ANN-PSO Models

GA is considered a useful method for solving optimization problems using the MATLAB 2015a software. PSO, as a new method, is able to accomplish the same goal as GA [39]. GA is inspired by the biological evolutionary process employing Darwin’s theory of survival of the fittest [40]. The optimization of GA begins with random solutions called population strings or chromosomes. Each of the chromosomes (combinations of four genes, *X*_1_, *X*_2_, *X*_3_, and *X*_4_) usually is represented as a binary string, which is evaluated by an objective function for their fitness [41]. In this process, GA uses three main types of rules (i.e., selection, crossover, and mutation) to create the new generation from the current population until one chromosome has the best fitness and thus is taken as the best solution to the problem. The values of the GA parameters for population size, number of generations, crossover rate, and mutation probability were 20, 100, 0.8, and 0.01.

PSO was firstly proposed by Kennedy and Eberhart. This metaheuristic method is inspired by the social behavior of birds flocking and fish schooling for searching for food [42]. PSO is started with a swarm of particles randomly positioned in multidimensional search space. Every particle is the solution to the problem, which has two characteristics, namely, velocity and position. These particles have a memory and it is helpful to keep track of its previous best position. The position corresponding to the best fitness is known as personal best and global best. After the completion of each iteration, the position of particles is adjusted based on its own historical behavior and neighbors. The particles continue to move in the search space until the maximum iteration number or the desired value of the objective function was reached. The operating parameters were the swarm size (20), maximum iteration (50), personal learning coefficient (2), global learning coefficient (2), minimum inertia weight (0.3), and maximum inertia weight (0.9).

## 3. Results and Discussion

### 3.1. Characterization of Fe/Ni Nanoparticles and rGO/Fe/Ni Composites

**Figure 4:**
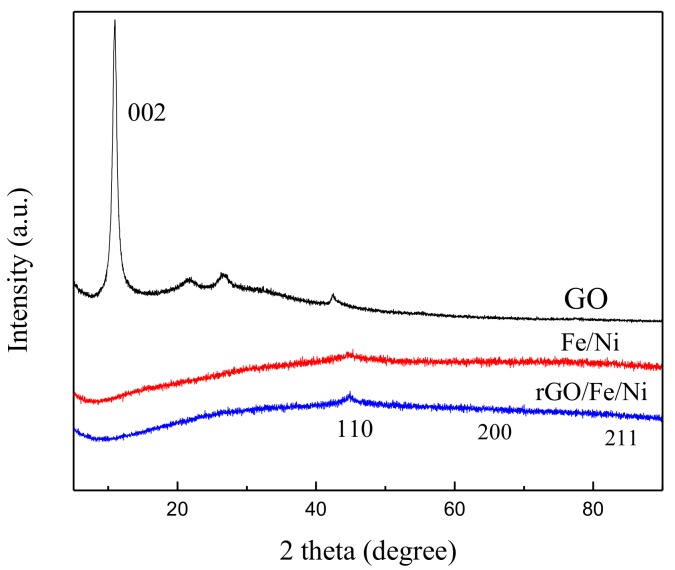
XRD patterns of GO, Fe/Ni, nanoparticles and reduced GO (rGO)/Fe/Ni composites.

The XRD patterns of the GO, Fe/Ni nanoparticles, and rGO/Fe/Ni composites are shown in Figure 4. The intense diffraction peaks of Fe^0^ at 2θ 44.4°, 58.7°, and 82.0° are assigned to the (110), (200), and (211) lattice planes, respectively. The prominent peak at 44.4° indicates the presence of Fe^0^ (JCPDS-00-001-01267) in Fe/Ni nanoparticles and rGO/Fe/Ni composites, which can be assigned to the (110) diffraction plane [43]. The diffraction peak of GO (10.9°, 001) in the pattern of rGO/Fe/Ni composites is not observed, demonstrating the reduction of GO. No diffraction peaks of Ni were observed, because of its low amounts in Fe/Ni nanoparticles and rGO/Fe/Ni composites.

**Figure 5:**
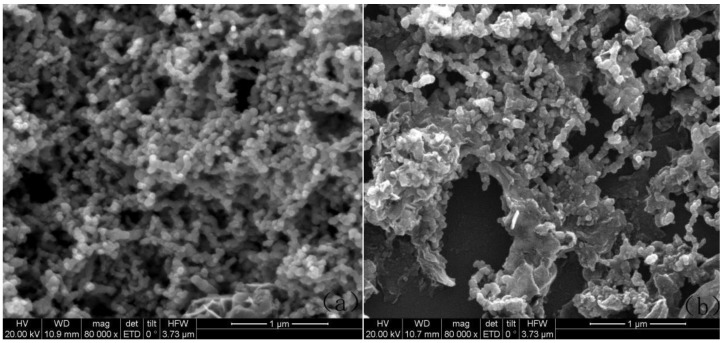
SEM images of Fe/Ni (**a**) and rGO/Fe/Ni (**b**).

**Figure 6:**
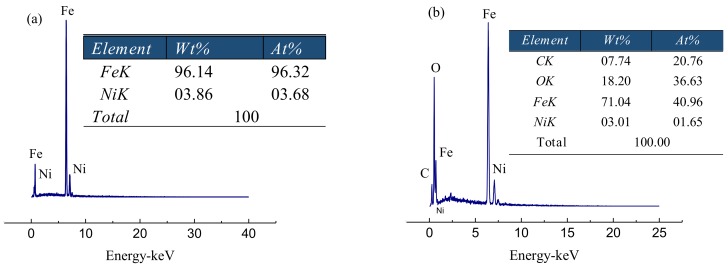
EDS spectra of Fe/Ni (**a**) and rGO/Fe/Ni (**b**).

**Figure 7:**
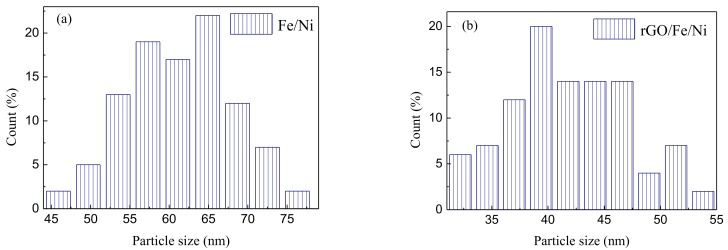
The distribution of diameters of Fe/Ni (**a**) and rGO/Fe/Ni (**b**).

The morphology of Fe/Ni and rGO/Fe/Ni was characterized by using SEM (Figure 5). Fe/Ni nanoparticles aggregated together tightly were observed (Figure 5a). As shown in Figure 5b, the Fe/Ni nanoparticles are homogeneously dispersed well on the rGO surface, which imply an inhibiting effect on the Fe/Ni nanoparticle aggregation. The spectra of EDS also illustrate the presence of Ni as shown in Figure 6a,b. nZVI was synthesized by Wang et al., and the average diameter of this material is 80 nm [44]. In this study, the particle sizes of Fe/Ni nanoparticles and rGO/Fe/Ni composites are 44–81 nm and 26–68 nm in diameter, and the average diameters of Fe/Ni nanoparticles and rGO/Fe/Ni composites are approximately 66 nm and 42 nm, respectively (Figure 7a,b). These results indicate that Ni can effectively prevent the aggregation of nZVI, and the Fe/Ni nanoparticles can be dispersed onto the rGO surface.

**Figure 8:**
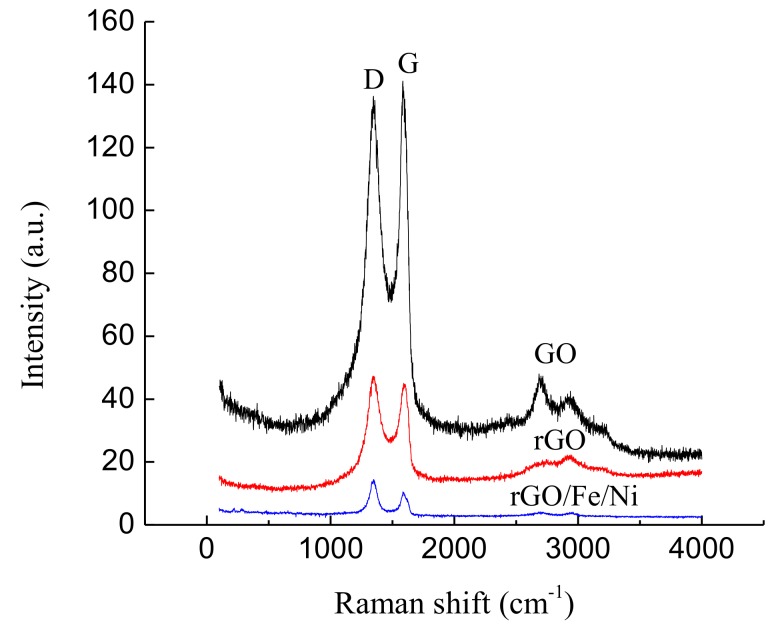
Raman spectra of GO, rGO, and rGO/Fe/Ni composites.

Raman spectroscopy can be used as an effective method to identify the degree of graphitization and structural changes in the GO-based nanomaterials. The spectra of the GO, rGO, and rGO/Fe/Ni are shown in Figure 8, which exhibit the characteristic D and G bands centered at 1345 and 1587 cm^−1^, respectively. The D band is a structural disorder originated from the breathing mode of A_1g_ symmetry, whereas the G band is of E_2g_ symmetry induced by the inplane vibrations of sp^2^ bond atoms [45]. The intensity ratio of the D to G bands (I_D_/I_G_) is often used as a measure of defect levels in graphene-based materials. The intensity of I_D_/I_G_ increases from 0.98 for GO to 1.07 for rGO and then to 1.38 for rGO/Fe/Ni composites. This phenomenon could be attributed to the decrease in the sp^2^ cluster size, perhaps caused by the creation of defects owing to the presence of iron atoms on the surface of the rGO.

**Figure 9:**
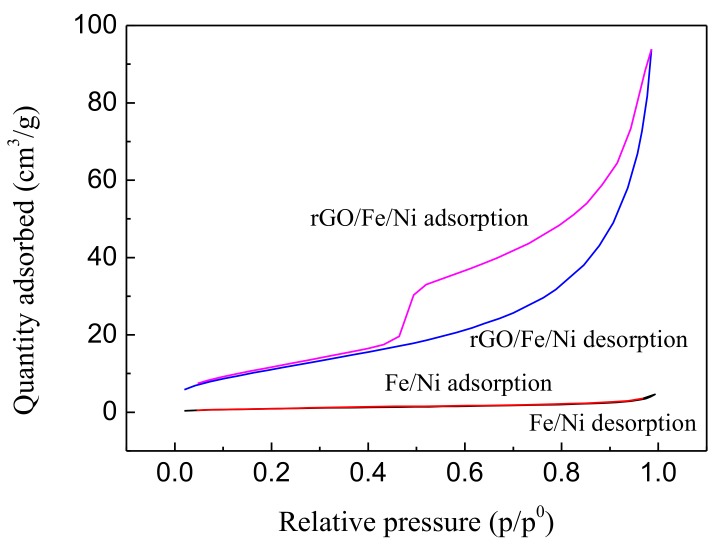
The adsorption/desorption of Fe/Ni nanoparticles and rGO/Fe/Ni composites.

The surface area of Fe/Ni nanoparticles and rGO/Fe/Ni composites was measured using a BET analyzer, and the surface area of these materials was calculated to be 3.70 and 43.31 m^2^/g (Figure 9). It is noted that the surface area of rGO/Fe/Ni composites is significantly higher than that of Fe/Ni nanoparticles. This is attributed to the satisfactory dispersion of Fe/Ni on the surface of rGO.

**Figure 10:**
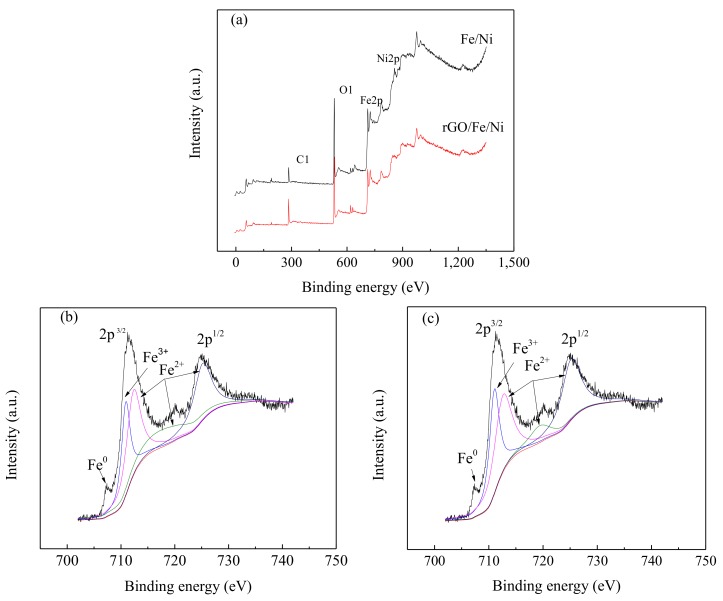
XPS analyses of Fe/Ni and rGO/Fe/Ni: wide scan (**a**); high-resolution spectra of Fe2p for Fe/Ni nanoparticles (**b**); and high-resolution spectra of Fe2p for rGO/Fe/Ni composites (**c**).

The results of X-ray photoelectron spectroscopy (XPS) show that the binding energies at 284.6, 554.6, 720.6, and 860.8 eV are attributed to C1s, O1s, Fe2p, and Ni2p, respectively (Figure 10a). The Fe2p spectra consist of 2p^1/2^ and 2p^3/2^ peaks, which are located at 710.55 and 725.37 eV. The binding energies of the shake-up satellite (2p^3/2^ and 2p^1/2^) at 719.94 eV and 724.88 eV indicate the existence of Fe^2+^ and Fe^3+^ [45]. A weak peak at 707.5 eV corresponding to Fe^0^ was observed in both the Fe/Ni and rGO/Fe/Ni (Figure 10b,c). The intensity of the Fe^0^ peak in the rGO/Fe/Ni is stronger than that of the Fe/Ni particles, indicating that rGO might decrease the oxidation degree of Fe/Ni.

### 3.2. The Zero Point of Charge of rGO/Fe/Ni Composites

**Figure 11:**
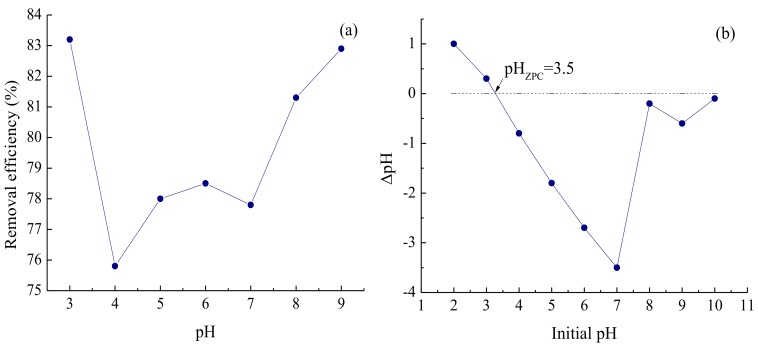
The relation between pH and the removal efficiency (**a**); the initial pH versus ΔpH (**b**).

The behavior of CV adsorption on rGO/Fe/Ni composites was studied over a broad range of pH (2–10). It is worth noting that an obvious increase in the removal efficiency of CV by rGO/Fe/Ni composites was observed with the increase in pH of the solution (Figure 11a). The pH_zpc_ value of this material is 3.5 (Figure 11b). For pH > pH_zpc_, the rGO/Fe/Ni composite surface will possess negative charge, which is in favor of the adsorption of CV [46,47]. For pH < pH_zpc_, this material’s surface is charged positively [48]. These results are in accordance with the effect of pH on the removal efficiency of CV.

### 3.3. Experimental Results

The BBD of RSM was applied to visualize the effect of various independent parameters, namely, initial pH, temperature, contact time, and initial concentration, on the dependent parameter (removal efficiency of CV). Experimental data and predicted values for the removal of CV from aqueous solution are listed in Table 3. A multivariate analysis was performed to describe the relationship between dependent parameters and independent parameters; the fitted model equation is shown as follows.

(11) $$Y=68.20-0.82X_{1}-5.48X_{2}+3.79X_{3}+3.13X_{4}+0.3X_{1}X_{2}-4.30X_{1}X_{3}+2.22X_{1}X_{4}-5.02X_{2}X_{3}+1.37X_{2}X_{4}-1.20X_{3}X_{4}+4.94X_{1}{}^{2}+2.85X_{2}{}^{2}+1.97X_{3}{}^{2}-1.47X_{4}{}^{2}$$

**Figure 12:**
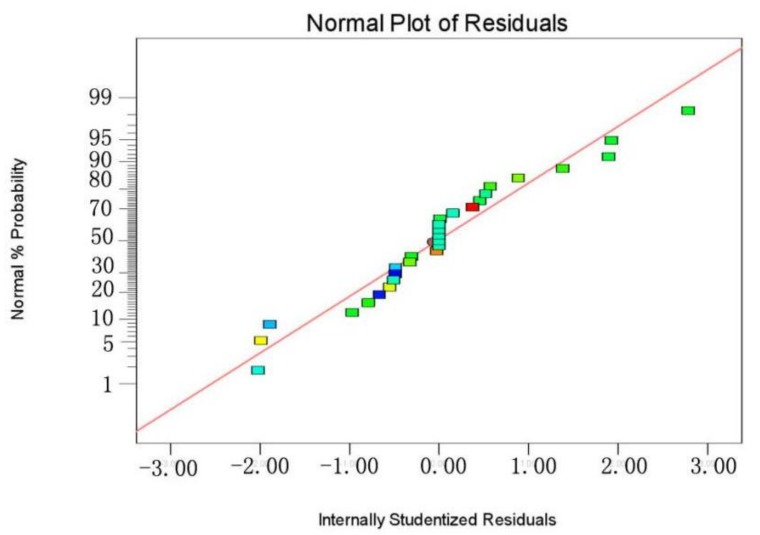
The normal probabilities versus internally studentized residuals.

**Figure 13:**
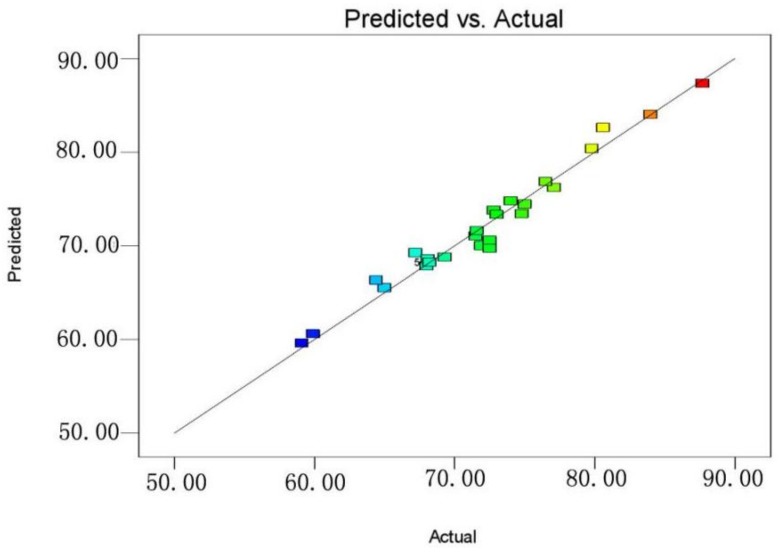
The predicted values versus the actual values.

**Figure 14:**
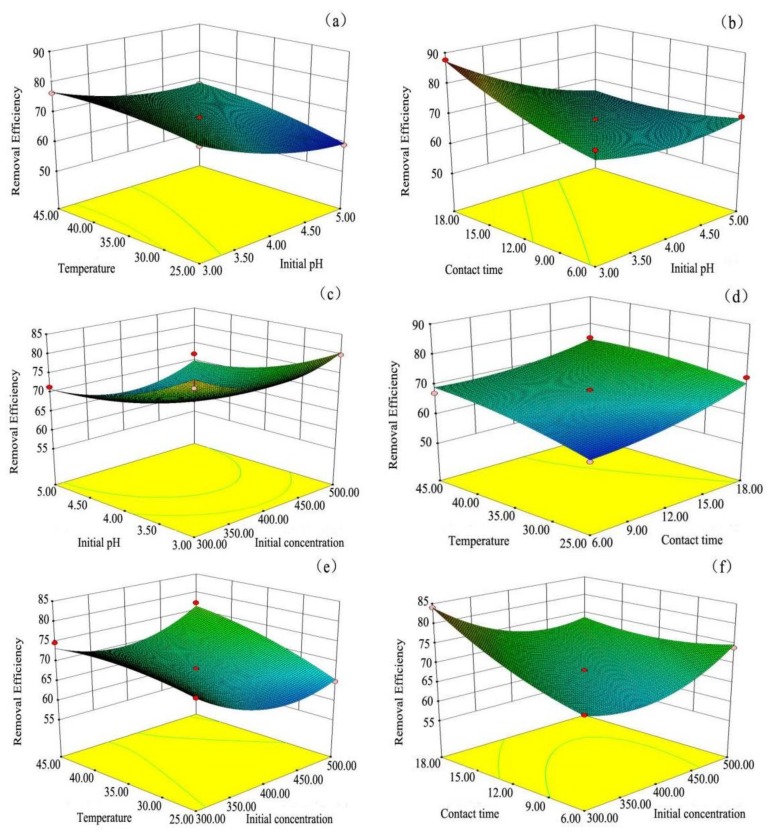
Three-dimensional response surface plots for the CV removal: (**a**) Initial pH–Temperature; (**b**) Initial pH–Contact time; (**c**) Initial pH–Initial concentration (**d**) Temperature–Contact time; (**e**) Temperature–Initial concentration; (**f**) Contact time–Initial concentration.

**Figure 15:**
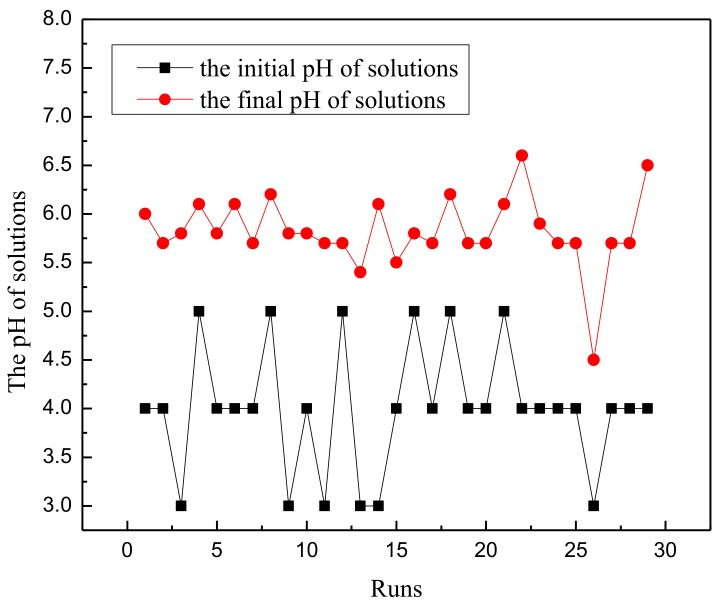
The difference between the initial and final pH of the solutions.

The plot of normal probabilities versus the residual values shows that the points of residuals on the plot follow a straight line, confirming the normality of the error distribution (Figure 12). The value of the determination coefficient (*R*^2^ = 0.9701) demonstrates that the predicted values were in agreement with experimental values (Figure 13). Three-dimensional response surface plots (a, b, c, d, e, and f) indicate the combined effect of initial pH and initial concentration, contact time and initial concentration, temperature and initial concentration, contact time and initial pH, temperature and initial pH, and temperature and contact time. It is evident that the maximum removal efficiency was recorded at high pH and low initial concentration (Figure 14). As shown in Figure 15, the value of final pH is higher than that of initial pH. This may be ascribed to the increased negative charge on the surface of rGO/Fe/Ni composites. Since the pH_zpc_ of rGO/Fe/Ni composites is 3.5, the adsorption of CV was also enhanced when pH > pH_zpc_.

Analysis of variance (ANOVA) was applied to examine the quality of the fitted model. If the values of *p* are less than 0.05, then the model terms have a statistically significant role on the CV removal (Table 4). Therefore, it can be concluded that the variables *X*_2_, *X*_3_, *X*_4_, *X*_1_*X*_3_, *X*_2_*X*_3_, and *X*_1_^2^ are all statistically significant model terms. The *F* values represent the significance of operating parameters on the CV removal by rGO/Fe/Ni composites, based on which the order for the importance of operating parameters is as follows: *X*_2_ > *X*_3_ > *X*_4_ > *X*_1_. This model has a good suitability due to its high *F*-values and nonsignificant lack of fit.

### 3.4. BP-ANN Model

**Figure 16:**
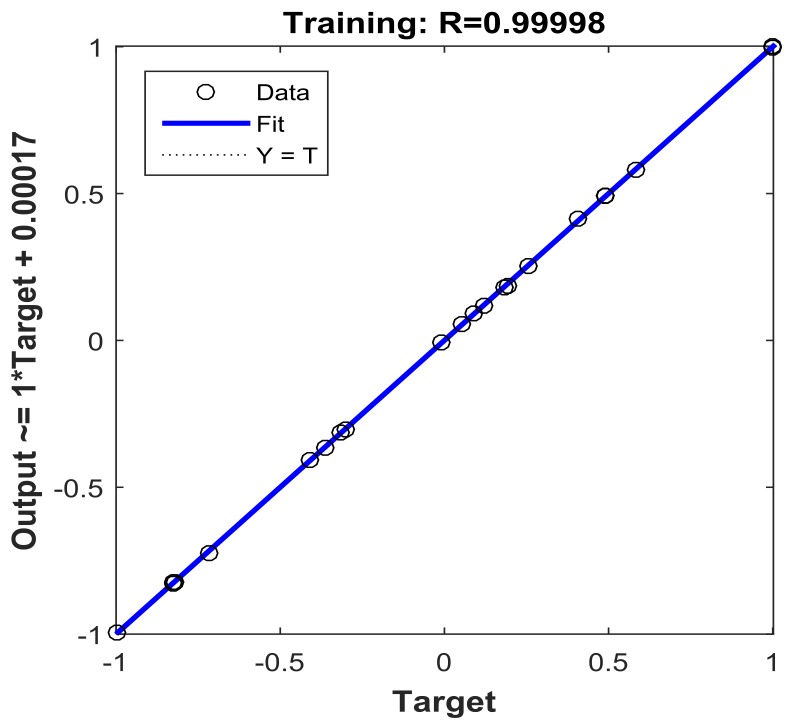
Regression plot of the experimental and predicted results.

**Figure 17:**
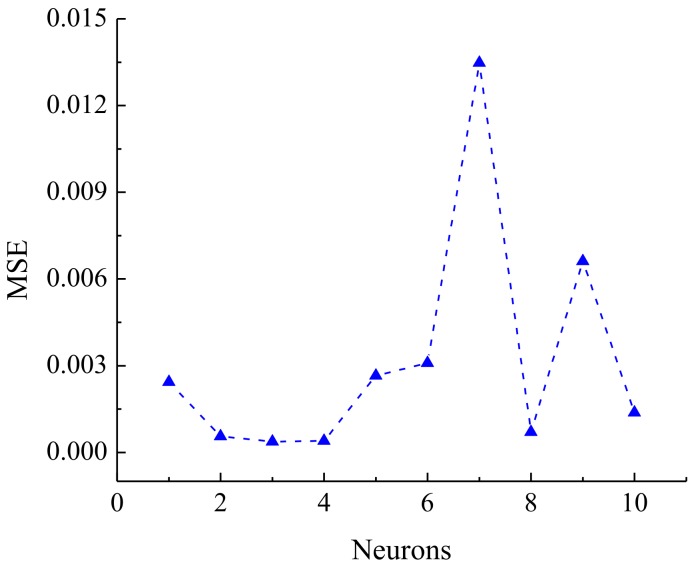
Mean square error (MSE) of neurons in the BP-ANN model.

**Figure 18:**
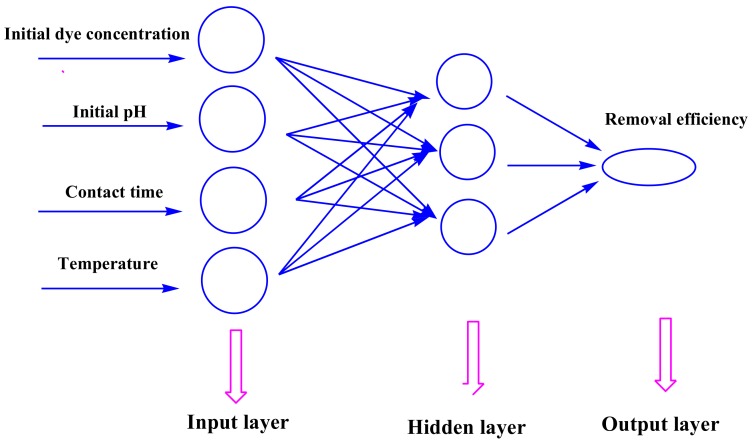
Applied BP-ANN model in this study.

The experimental data used in the BP-ANN model were collected in this study from BBD. Of the whole data set, 80% (1–24) was used for training and 20% (25–29) was used for testing (Table 5). The value of *R*^2^ (0.9998) of the BP-ANN model indicates the best prediction ability for testing and training in this network (Figure 16). The number of neurons (N) in the hidden layer was determined according to the minimum mean square error (MSE) of the neural network. In order to determine the optimum number of neurons in the hidden layer, the number of nodes was examined by varying from 1 to 10. The values of MSE were used as the error function (Figure 17). As can be seen in Figure 18, the ANN architecture with 3 neurons in the hidden layer appeared to be the optimal topology for training. The influence for each input variable on the output variable was calculated by the Garson equation using the weight and bias (Table 6). It was reported that temperature gives the highest percentage contribution to the decolorization of CV with 36.27% followed by 20.23% of initial concentration, 35.95% of initial pH, and 7.54% of contact time (Table 7).

### 3.5. Prediction by BBD, ANN-PSO, and ANN-GA

**Figure 19:**
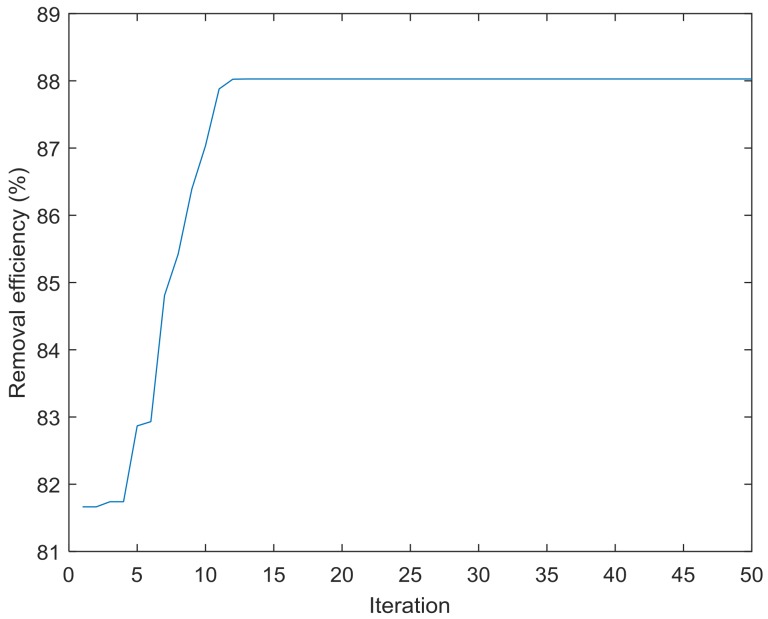
Removal efficiency versus iteration.

**Figure 20:**
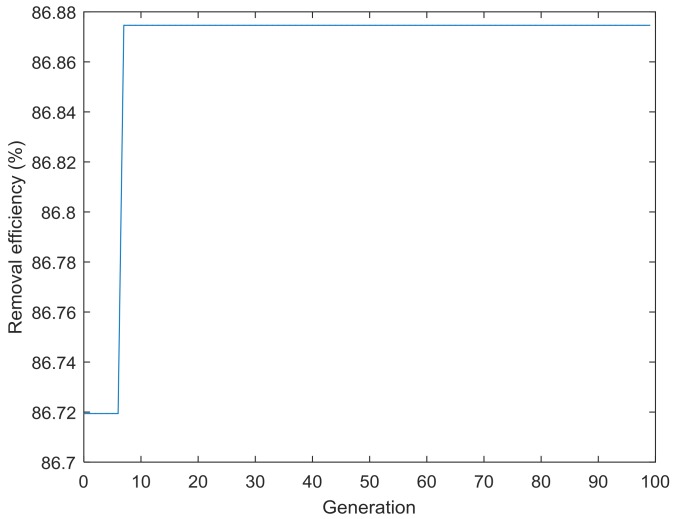
Removal efficiency versus generation.

The optimum values of independent parameters for BBD are 3.0 for pH, 40.6 °C for temperature, contact time of 18.0 min, and initial concentration of 368.9 mg/L. The maximum removal efficiency predicted under this condition was 88.2%, while the corresponding experimental value was 75.8% (Table 8). The maximum percentage decolorization predicted by using ANN-PSO model is 88.0%, and the corresponding experimental value is 84.5% (Figure 19). The performance of the ANN-GA model indicates that the prediction removal efficiency is 86.9% under the following condition: pH of 3.3, temperature of 36.1 °C, contact time of 15.8 min, and initial concentration of 331.2 mg/L (Figure 20). The absolute errors between the predicted and experimental results are 12.4, 3.5, and 5.6 for the BBD, ANN-PSO, and ANN-GA models, respectively. It was found that ANN-PSO was suitable for the prediction of CV removal by rGO/Fe/Ni composites.

### 3.6. Isotherm Studies

The adsorption data for the CV onto rGO/Fe/Ni nanocomposites were fitted to Langmuir, Freundlich, Temkin, and Dubinin–Radushkevich (D-R) models. The isotherm studies were performed under contact time of 18 min, pH of 5, and temperature of 25 °C with initial dye concentration of CV ranging from 200 to 1000 mg/L in order to provide an insight into the adsorption characteristics of rGO/Fe/Ni composites. The Freundlich isotherm assumes that the adsorption occurs on a heterogeneous surface with a multilayer adsorption mechanism, which is expressed as follows [49]:

(12) $$\log q_{e}=\log k_{f}+1/n\log c_{e}$$

where *q_e_* is the amount of CV adsorbed (mg/g); *c_e_* is the equilibrium concentration of CV (mg/L); and *K_f_* and *n* are constants affecting the adsorption capacity and intensity of adsorption, respectively, with value closer to zero with the rising heterogeneous nature of the surface (1/*n* < 1 indicates normal Langmuir isotherm while 1/*n* above 1 indicates bimechanism and cooperative adsorption).

The Langmuir isotherm describes the adsorption process occurring in a single layer (forming a molecular monolayer) of the adsorbent surface, and was employed for the calculation of the maximum capacity of adsorption (*q_max_*). A plot of 1/*q_e_* versus 1/*c_e_* was used to obtain the Langmuir equilibrium isotherm. The Langmuir isotherm equation in linear form is given as follows [50]:

(13) $$c_{e}/q_{e}=1/q_{m}K_{L}+c_{e}/q_{m}$$

(14) $$R_{L}=1/1+K_{L}c_{0}$$

where *c*_0_ and *c_e_* are the initial and final dye concentration (mg/L), respectively; *q_e_* is the amount of CV at equilibrium (mg/g); *q_m_* is the maximum adsorption capacity; *K_L_* is the Langmuir constant (L/mg); and *R_L_* is the separation factor that indicates the adsorption nature to be either unfavorable (*R_L_* > 1), linear (*R_L_* = 1), favorable (0 < *R_L_* < 1), or irreversible (*R_L_* = 0) [51].

The Temkin isotherm considers that the heat of adsorption for all molecules in the phase will decrease linearly with coverage owing to adsorbent–adsorbate interaction [52,53].

(15) $$q_{e}=B\ln A+B\ln c_{e}$$

where *B* is related to the heat of adsorption, while *A* (L/g) is the binding constant responding to the maximum binding energy at equilibrium; *c_e_* and *q_e_* have the same meanings as mentioned above.

The Dubinin–Radushkevich (D-R) isotherm was applied to estimate whether the adsorption process has a physical or chemical mechanism [54,55]. The linear form of the D-R isotherm is presented as the following equations [56,57]:

(16) $$\ln q_{e}=\ln q_{\max}-\alpha \varepsilon^{2}$$

(17) $$\varepsilon =RT\ln\left(1+1/c_{e}\right)$$

(18) $$E=2\alpha^{-1/2}$$

where *c_e_*, *q_e_*, and *q_max_* have the same meanings as mentioned above; *T* is the absolute solution temperature (K); *R* is the universal gas constant, 8.314 (J/mol K); α is a constant energy (mol^2^/J^2^); and ε is the Polanyi potential. The value of *E* gives information about the adsorption mechanism: physical or chemical. If it lies between 8 and 16 kJ/mol, the adsorption process is controlled by a chemical mechanism, while a value of *E* smaller than 8 kJ/mol indicates that the adsorption process was physisorption [58].

The calculated isotherm parameters for the adsorption of CV onto rGO/Fe/Ni composites are presented in Table 9. The value of *K_F_* for the Freunlich model was 98.7 and the 1/*n* was found to be 0.4572, demonstrating that the adsorption of CV onto the rGO/Fe/Ni composites was a favorable process. The values of *R_L_* (7.8 × 10^−6^–3.9 × 10^−5^) are in the range of 0–1, indicating that the process is favorable, which corroborates the *K_F_* values of the Freunlich isotherm. The value of the Langmuir isotherm constant (*K_L_*) is 128.8 L/mg. The maximum adsorption capacity from the Langmuir isotherm was calculated to be 2000 mg/g. The mean adsorption energy value of CV was 0.2 KJ/mol, implying that the adsorption of CV by rGO/Fe/Ni composites was mostly chemisorption. The value of *R*^2^ for the Freundlich isotherm (0.9700) was higher than those for the Langmuir (0.9483), Temkin (0.9094), and D-R (0.7339) isotherms. The values of the adsorption isotherm constants *n* and *R*^2^ indicate that the experimental values are well fitted to the Freundlich model. As given in Table 10, the removal capacity of the rGO/Fe/Ni composites is significantly higher than that of other materials. The excellent CV removal capacity of rGO/Fe/Ni composites is an important advantage for environmental remediation.

### 3.7. Kinetics Studies

A study on the kinetics was applied to describe the solute uptake rate and the rate-controlling step of the process for the CV adsorption onto rGO/Fe/Ni composites. The experimental data were fitted to the pseudo-first-order, pseudo-second-order, intraparticle diffusion, and Elovich kinetic models.

The pseudo-first-order kinetic model is presented by the relation of Lagergren, which states that the adsorption rate of vacant sites is proportional to the number of occupied sites, and the equations can be described by the following form [64,65]:

(19) $$\log\left(q_{e}-q_{t}\right)=loq_{e}-k_{1}t/2.303$$

where *q_e_* (mg/g) and *q_t_* (mg/g) are the adsorption capacity at equilibrium and at time *t* (min), respectively, and *k*_1_ (min/1) is the first-order rate constant. The pseudo-second-order model can be represented in the following form [66]:

(20) $$t/q_{t}=1/k_{2}q_{e}{}^{2}+t/q_{e}$$

where *k*_2_ is the pseudo-second-order rate constant (g/mg/min), and the adsorption rate constants (*k*_2_) can be determined experimentally by plotting of *t*/*q_t_* versus *t*.

To study the adsorption mechanism, the kinetic data of adsorption were fitted into intraparticle diffusion and Elovich equations. The intraparticle diffusion model was described by Weber and Morriss, and can be used to investigate the rate-limiting step for CV adsorption onto rGO/Fe/Ni composites [67,68]:

(21) $$q_{t}=k_{3}t^{0.5}+B$$

where *k*_3_ is the rate constant of intraparticle diffusion (mg/g min^1/2^), and the value of *B* is proportional to the boundary layer [69].

The Elovich model implies multilayer adsorption and assumes that the adsorption sites increase exponentially during the adsorption process; this equation can be written as follows [70]:

(22) $$q_{t}=1/\beta \ln\left(\alpha \beta \right)+1/\beta \ln t$$

where *α* (mg/g/min) is the initial adsorption rate and *β* (g/mg) is the desorption constant, which is related to the extent of surface coverage and activation energy for chemisorption.

The values of parameters obtained from these models are presented in Table 11. From the values of *R*^2^, it can be concluded that the experimental data are well fitted to the pseudo-second-order kinetic model in contrast with the pseudo-first-order, intraparticle diffusion, and Elvoich kinetics. This implicates that the adsorption is of a chemical nature.

### 3.8. Thermodynamics Studies

In order to investigate the effect of temperature on the adsorption of CV, experiments were carried out at four different temperatures: 298, 308, 318, and 328 K. The amount of adsorption was increased from 946.21 to 1087.04 mg/g with the increase in temperature. Kc was calculated using the following equation [71]:

(23) $$K_{c}=q_{e}/c_{e}.$$

The enthalpy change Δ*H*^0^ (kJ/mol) and entropy change Δ*S*^0^ (J/mol/K) for the adsorption of CV were calculated from the slope and intercept of the plot of ln(*K_c_*) v/s 1/*T*, and these parameters can be obtained from the Van’t Hoff equation [72]:

(24) $$\ln K_{c}=\Delta S^{0}/R-\Delta H^{0}/RT.$$

The values of Gibbs free energy (Δ*G*^0^) was computed from the following equation:

(25) $$\Delta G^{0}=\Delta H^{0}-T\Delta S^{0}.$$

**Figure 21:**
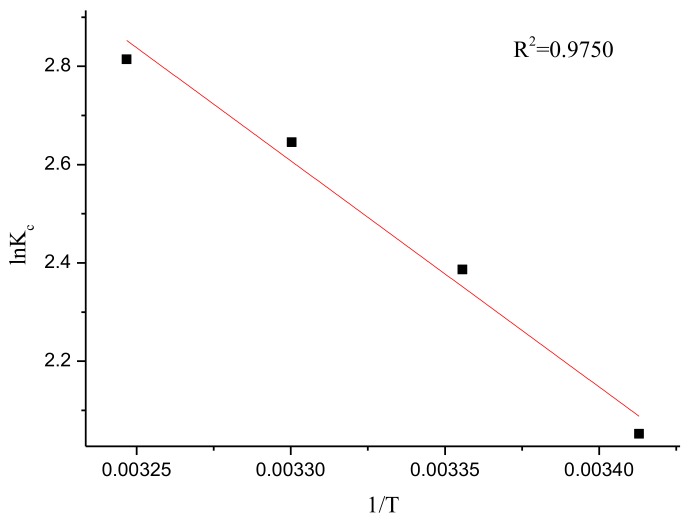
Plot of ln*K_c_* versus 1/*T* for initial pH of 5, contact time of 18 min, and initial concentration of 500.00 mg/L.

Values of Δ*G*^0^ for the adsorption of CV onto rGO/Fe/Ni composites in this work were found to be −5.0850, −5.9120, −6.5544, and −6.9719 kJ/mol at 293, 303, 313, and 323 K, respectively (Table 12). The negative values of Δ*G*^0^ demonstrate the spontaneous nature of the adsorption process. In addition, by plotting a graph of ln *K_c_* versus 1/*T*, the values of Δ*H*^0^ and Δ*S*^0^ can be obtained from the slopes and intercepts (Figure 21). The positive Δ*H*^0^ value suggests the endothermic nature of the adsorption process that is supported by the increase in removal efficiency with increasing temperature [73]. A positive value of entropy change was observed, which corresponded to the increase of randomness at the solid/liquid interface in the adsorption process [74]. The positive values of Δ*S*^0^ and Δ*H*^0^ show that the interaction of rGO/Fe/Ni composites with CV is basically entropy driven [75].

## 4. Conclusions

The results of this study demonstrate that rGO/Fe/Ni composites can be used effectively for the removal of CV from aqueous solutions. This material was synthesized by using the co-precipitation method, and characterized by XRD, SEM, EDS, Raman spectroscopy, N_2_-sorption, and XPS. Operating parameters such as initial concentration, initial pH, contact time, and temperature were investigated with the aid of BBD, ANN-GA, and ANN-PSO models to predict the optimum conditions and the maximum removal efficiency. The high value of *R*^2^ (0.9998) for the BP-ANN model indicates the best prediction ability. The absolute errors between predicted and experimental results were 12.4 for BBD, 5.6 for ANN-GA, and 3.5 for ANN-PSO. The existence of the high degree of agreement between the predicted results and experimental results indicate that the ANN-PSO could be used effectively for the evaluation and optimization of the effects of the independent variables on the removal efficiency of CV. Since the pH_zpc_ of rGO/Fe/Ni composites is 3.5, the adsorption of CV was enhanced when pH > pH_zpc_. The maximum adsorption capacity from the Langmuir isotherm was calculated to be 2000 mg/g. The value of *R*^2^ for the Freundlich isotherm (0.9700) was higher than those for the Langmuir (0.9483), Temkin (0.9094), and D-R (0.7339) isotherms. The experimental data is well fitted to the pseudo-second-order kinetic model. The negative values of Δ*G*^0^ illustrate the spontaneous nature in the range of temperature studied (293–323 K). The positive values of Δ*S*^0^ and Δ*H*^0^ show that the interaction of rGO/Fe/Ni composites with CV is basically entropy driven. Further studies should be performed concerning the zeta potential and the oxidation–reduction potential of nanomaterials, as well as the removal of two dyes simultaneously.

## Figures and Tables

**Table 1:** Characteristics of CV dye.

Chemical Name	Crystal Violet
Molecular formula	C_25_H_30_N_3_Cl
Molecular weight	408 g/mol
Maximum wavelength λ	583 nm

**Table 2:** Independent variables and levels used for the removal of CV.

Independent Variables	Factors	Levels		
		−1	0	1
Initial dye concentration (mg/L)	*X*_1_	300	400	500
Initial pH	*X*_2_	3	4	5
Contact time (min)	*X*_3_	6	12	18
Temperature (°C)	*X*_4_	25	35	45

**Table 3:** Comparison between predicted removal efficiency by the Box–Behnken design (BBD) model and experimental values.

Run	*X*_1_ (mg/L)	*X*_2_	*X*_3_ (min)	*X_4_* (°C)	Actual (%)	Predicted (%)
1	300	4	12	25	71.6	71.5
2	400	4	12	35	68.2	68.2
3	300	3	12	35	80.6	82.6
4	300	5	12	35	71.5	71.0
5	500	4	6	35	74	74.8
6	400	4	6	25	59.9	60.6
7	500	4	12	45	77.1	76.2
8	400	5	6	35	69.3	68.8
9	400	3	18	35	87.7	87.3
10	400	4	18	45	75	74.4
11	500	3	12	35	79.8	80.4
12	500	5	12	35	71.9	70.0
13	400	3	12	25	73	73.3
14	400	3	6	35	72.5	69.7
15	500	4	18	35	72.8	73.8
16	400	5	18	35	64.4	66.3
17	500	4	12	25	65	69.6
18	400	5	12	45	68.1	68.6
19	300	4	12	45	74.8	73.4
20	400	4	12	35	68.2	68.2
21	400	5	12	25	59.1	59.6
22	400	4	6	45	67.2	69.2
23	300	4	18	35	84	84.2
24	400	4	12	35	68.2	68.2
25	400	4	18	25	72.5	70.1
26	400	3	12	45	76.5	76.8
27	400	4	12	35	68.2	68.2
28	400	4	12	35	68.2	68.2
29	300	4	6	35	68	67.8

**Table 4:** Analysis of variance (ANOVA) for the experimental results from the response surface methodology (RSM).

Source	Sum of Squares	Degree of Freedom	Mean Square	*F*-Value	*p*-Value	
Model	1110.15	14	79.30	32.49	<0.0001	Significant
*X*_1_	8.17	1	8.17	3.35	0.0887	
*X*_2_	360.80	1	360.80	147.82	<0.0001	
*X*_3_	172.52	1	172.52	70.68	<0.0001	
*X*_4_	117.81	1	117.81	48.27	<0.0001	
*X*_1_ *X*_2_	0.36	1	0.36	0.15	0.7067	
*X*_1_ *X*_3_	73.96	1	73.96	30.30	<0.0001	
*X*_1_ *X*_4_	19.80	1	19.80	8.11	0.0129	
*X*_2_ *X*_3_	101.00	1	101.00	41.38	<0.0001	
*X*_2_ *X*_4_	7.56	1	7.56	3.10	0.1002	
*X*_3_ *X*_4_	5.76	1	5.76	2.36	0.1468	
*X*_1_2	158.40	1	158.40	64.90	<0.0001	
*X*_2_2	52.84	1	52.84	21.65	0.0004	
*X*_3_2	25.09	1	25.09	10.28	0.0063	
*X*_4_2	14.03	1	14.03	5.75	0.0310	
Residual	107.84	14	7.70	-	-	
Lack of Fit	107.84	10	10.78	-	-	
Pure Error	0	4	0	-	-	
Total	5777.37	28	-	-	-	

**Table 5:** Comparison of the predicted values by using the back-propagation (BP)-artificial neural network (ANN) model with the experimental values.

Runs	*X*_1_ (mg/L)	*X*_2_	*X*_3_ (min)	*X*_4_ (°C)	Experimental Value (%)	Predicted Value (%)	Absolute Error (%)
1	300	4	12	25	71.6	71.45	0.15
2	400	4	12	35	68.2	68.12	0.08
3	300	3	12	35	80.6	80.5	0.1
4	300	5	12	35	71.5	71.4	0.10
5	500	4	6	35	74	74.02	0.02
6	400	4	6	25	59.9	59.99	0.09
7	500	4	12	45	77.1	77.03	0.07
8	400	5	6	35	69.3	69.29	0.01
9	400	3	18	35	87.7	86.80	0.90
10	400	4	18	45	75	74.98	0.02
11	500	3	12	35	79.8	79.92	0.12
12	500	5	12	35	71.9	71.88	0.02
13	400	3	12	25	73	73.42	0.12
14	400	3	6	35	72.5	72.53	0.03
15	500	4	18	35	72.8	72.78	0.02
16	400	5	18	35	64.4	64.35	0.05
17	500	4	12	25	65	65.08	0.08
18	400	5	12	45	68.1	67.9	0.02
19	300	4	12	45	74.8	74.88	0.08
20	400	4	12	35	68.2	68.23	0.03
21	400	5	12	25	59.1	59.15	0.05
22	400	4	6	45	67.2	67.34	0.14
23	300	4	18	35	84	84.06	0.06
24	400	4	12	35	68.2	68.38	0.18
25 *	400	4	18	25	72.5	72.49	0.01
26 *	400	3	12	45	76.5	76.68	0.12
27 *	400	4	12	35	68.2	68.16	0.04
28 *	400	4	12	35	68.2	68.17	0.03
29 *	300	4	6	35	68	67.03	0.03
Mean absolute error (%)					0.10		

* representation test set.

**Table 6:** The weights and biases of BP-ANN in input–hidden layers (*w_i_* and *b_i_*) and hidden–output layer (*w_j_* and *b_j_*).

Number of Neurons	*w_i_*				*b_i_*	*w_j_*	*b_j_*
	Input Variables						
	Initial Dye Concentration	Initial pH	Contact Time	Temperature			
1	−0.05183	0.2899	−1.9836	−1.4754	−2.4896	−0.9964	0.2743
2	1.0057	−0.04558	1.1144	1.9856	1.9363	0.4235	
3	−1.4023	−1.0854	−1.7355	0.2040	1.3831	0.7355	
4	−1.1128	1.92090	−0.5651	−0.975	−0.8299	−0.7634	
5	2.0220	−1.3428	−0.4420	0.3335	0.2766	−0.9220	
6	0.3420	1.3265	1.4140	1.5239	0.2766	0.1964	
7	−0.6076	−1.4517	−1.4871	1.2288	−0.8299	0.2086	
8	1.9255	−0.6918	1.4166	−0.07202	−1.3831	0.03286	
9	1.9083	1.1988	−1.0214	−0.2760	1.9363	−0.9850	
10	0.7757	−1.0114	−1.3414	−1.6655	2.4896	0.3779	

**Table 7:** Relative influence of input variables.

Input Variables	Relative Significance (%)	Order
Initial dye concentration	20.23%	3
Initial pH	35.95%	2
Contact time	7.54%	4
Temperature	36.27%	1

**Table 8:** Comparison between the predicted percentage decolorization of CV by using BBD, ANN-particle swarm optimization (PSO), and ANN-genetic algorithm (GA) models and the experimental results.

Models	Independent Parameters				Prediction (%)	Experiment (%)	Absolute Error (%)
	*X_1_*	*X_2_*	*X_3_*	*X_4_*			
BBD	368.9	3.0	18.0	40.6	88.2	75.8	12.4
ANN-PSO	300.0	4.0	18.0	45.0	88.0	84.5	3.5
ANN-GA	331.2	3.3	15.8	36.1	86.9	81.3	5.6

**Table 9:** Isotherm parameters for the adsorption of CV onto the rGO/Fe/Ni composites.

Isotherm Models	Parameters	Value of Parameters
Freundlich	*K_f_* (mg/g)	98.7
	1/*n*	0.4572
	*R* ^2^	0.9700
Langmuir	*K_L_* (L/mg)	128.8
	*q_m_* (mg/g)	2000.00
	*R* ^2^	0.9483
	*R_L_*	7.8 × 10^−6^–3.9 × 10^−5^
Temkin	*A* (L/g)	0.089
	*B*	402.97
	*R* ^2^	0.9094
Dubinin–Radushkevich	*q_m_* (mg/g)	1350.4
	α (mol^2^/J^2^)	1.0 × 10^−3^
	*E* (kJ/mol)	0.2
	*R* ^2^	0.7339

**Table 10:** CV adsorption capacity of the rGO/Fe/Ni composites and other materials.

Adsorbents	Maximum Adsorption Capacity (mg/g)	References
Chitosan–graphite oxide	64.935	[59]
NaOH-treated almond shell	123	[60]
Surfactant-modified alumina	254	[61]
Grafted polyacrylamide on SiO_2_ nanocomposites	378.8	[62]
Fe_3_O_4_/porous graphene	460	[63]
rGO/Fe/Ni composites	2000	This study

**Table 11:** Kinetic parameters for the adsorption of CV onto the rGO/Fe/Ni composites.

Kinetic Models	Parameters	Values of Parameters
Pseudo-first-order	*k*_1_ (1/min)	0.18
	*q_e_* (mg/g)	953.89
	*R* ^2^	0.9091
Pseudo-second-order	*k*_2_ (g/mg/min)	2.0 × 10^−3^
	*q*_e_ (mg/g)	1111.11
	*R* ^2^	0.9946
Intraparticle diffusion	*k*_3_(mg/g/min^1/2^)	128.04
	*B* (mg/g)	364.18
	*R* ^2^	0.957
Elovich	*α* (mg/g/min)	733.34
	*β* (g/mg)	4.5 × 10^−2^
	*R* ^2^	0.9724

**Table 12:** The thermodynamic parameters for the adsorption of CV onto rGO/Fe/Ni composites.

K	Δ*G*^0^ (KJ/mol)	Δ*S*^0^ (kJ/mol/K)	Δ*H*^0^ (kJ/mol)
293	−5.0850	0.1480	38.2450
303	−5.9120		
313	−6.5544		
323	−6.9719		
